# Progressive Volume Loss and White Matter Degeneration in Cstb-Deficient Mice: A Diffusion Tensor and Longitudinal Volumetry MRI Study

**DOI:** 10.1371/journal.pone.0090709

**Published:** 2014-03-06

**Authors:** Otto Manninen, Teemu Laitinen, Kimmo K. Lehtimäki, Saara Tegelberg, Anna-Elina Lehesjoki, Olli Gröhn, Outi Kopra

**Affiliations:** 1 Folkhälsan Institute of Genetics, Helsinki, Finland; 2 Department of Medical Genetics and Research Program's Unit Haartman Institute, University of Helsinki, Helsinki, Finland; 3 Neuroscience Center, University of Helsinki, Helsinki, Finland; 4 Department of Neurobiology A. I. Virtanen Institute for Molecular Sciences, University of Eastern Finland, Kuopio, Finland; University of Pécs Medical School, Hungary

## Abstract

Unverricht-Lundborg type progressive myoclonus epilepsy (EPM1, OMIM 254800) is an autosomal recessive disorder characterized by onset at the age of 6 to 16 years, incapacitating stimulus-sensitive myoclonus and tonic-clonic epileptic seizures. It is caused by mutations in the gene encoding cystatin B. Previously, widespread white matter changes and atrophy has been detected both in adult EPM1 patients and in 6-month-old cystatin B–deficient mice, a mouse model for the EPM1 disease. In order to elucidate the spatiotemporal dynamics of the brain atrophy and white matter changes in EPM1, we conducted longitudinal *in vivo* magnetic resonance imaging and *ex vivo* diffusion tensor imaging accompanied with tract-based spatial statistics analysis to compare volumetric changes and fractional anisotropy in the brains of 1 to 6 months of age cystatin B–deficient and control mice. The results reveal progressive but non-uniform volume loss of the cystatin B–deficient mouse brains, indicating that different neuronal populations possess distinct sensitivity to the damage caused by cystatin B deficiency. The diffusion tensor imaging data reveal early and progressive white matter alterations in cystatin B–deficient mice affecting all major tracts. The results also indicate that the white matter damage in the cystatin B–deficient brain is most likely secondary to glial activation and neurodegenerative events rather than a primary result of CSTB deficiency. The data also show that diffusion tensor imaging combined with TBSS analysis provides a feasible approach not only to follow white matter damage in neurodegenerative mouse models but also to detect fractional anisotropy changes related to normal white matter maturation and reorganisation.

## Introduction

Unverricht-Lundborg disease, also known as a progressive myoclonus epilepsy type 1 (EPM1, OMIM 254800), is an autosomal recessive neurodegenerative disorder and the most common cause of progressive myoclonus epilepsy. EPM1 is characterized by onset at age of 6–16 years and the symptoms include stimulus-sensitive myoclonus, tonic-clonic epileptic seizures and ataxia [1]. EPM1 is most commonly caused by a homozygous dodecamer repeat expansion mutation in the promoter region of the cystatin B (*CSTB*) gene, although thirteen other disease-causing mutations in *CSTB* are currently known [2]–[5]. The causative mutations lead to reduced expression of the cystatin B (CSTB) protein [6], [7] that is a ubiquitously expressed inhibitor of lysosomal cysteine cathepsins B, H, K, L, and S. Despite the fact that the causative gene mutations for EPM1 are known, the underlying mechanisms leading to the characteristic symptoms of the disease remain elusive. Although EPM1 patients do not show major cognitive decline, histopathological and imaging studies have confirmed atrophic changes on several brain regions of adult EPM1 patients, affecting both grey matter (GM) and white matter (WM) [1], [8], [9].

A mouse model for EPM1 has been generated with a targeted disruption of the mouse *Cstb* gene (the *Cstb^-/-^* mouse) and it recapitulates the key symptoms of EPM1, including myoclonic seizures and progressive ataxia [10]. The findings in adult *Cstb^-/-^* mice are compatible with the neuropathology found in patients, and exhibit neuronal death in the cerebrum and cerebellum, degenerative changes in the WM, and loss in brain volume [9]–[13]. In order to clarify the spatiotemporal progression of the brain pathology, this study aims to quantify the dynamics of brain atrophy and WM changes in *Cstb^-/-^* mice from 1 month of age up to the fully symptomatic age of 6 months using *in vivo* magnetic resonance imaging (MRI) for detecting volumetric changes and *ex vivo* diffusion tensor imaging (DTI) with tract-based spatial statistics (TBSS) analysis for detecting changes in WM integrity [14].

## Materials and Methods

### 2.1 Ethics statement

All the animal research protocols were approved by the Animal Ethics Committee of the State Provincial Office of Southern Finland (decisions ESLH-2005-00507/Ym-23, ESLH-2007-05788/Ym-23, ESAVI-2010-07744/Ym-23).

### 2.2 Mice

*Cstb^-/-^* mice (129-*Cstb* ^tm1Rm^/SvJ; stock no. 003486; Jackson Laboratories) between 1 to 6 months of age were used in the study and wild-type animals of the same background served as controls. Animals were maintained under a 12-h light/12-h dark cycle with lights on at 07:00 a.m., temperature 22±1°C, air humidity 50–60%, and with free access to food and water.

### 2.3 Magnetic resonance imaging

*In vivo* MRI was performed on *Cstb^-/-^* and control mice (*Cstb^-/-^*; n = 4, all females/controls; n = 4, all females) at 1, 2, 4, and 6 months of age. Mice were anesthetized with 1% isoflurane in 70% N_2_O/30% O_2_, and the body temperature was maintained at 37°C during imaging. MRI was performed using a 4.7 T Varian UNITY INOVA MRI system (Varian Inc., Palo Alto, USA) with an actively decoupled volume coil-quadrature surface coil pair (Rapid Biomedical GmbH, Rimpar, Germany). A multi-slice (17 slices) spin echo sequence was used to acquire anatomical images for volumetric analysis with the following parameters; 100×100 µm in-plane resolution; matrix 256×256, slice thickness 0.75 mm, echo time (TE) 40 ms, repetition time (TR) 1.8 s. Regions of interest (ROIs) were drawn on blinded data sets according to Paxinos mouse brain atlas [15] for the whole brain (excluding the medulla), the cerebellum, cerebral cortices, striatum and hippocampus for each time point using in-house built software Aedes 1.0 (aedes.uef.fi). For statistical comparisons, we performed one-way ANOVA with Bonferroni post hoc correction using GraphPad (Prism 5.0c; GraphPad Software, La Jolla, USA) and considered p<0.05 as significant.

### 2.4 Diffusion tensor imaging

For the *ex vivo* brain DTI, mice that were 2 months old (9 *Cstb^-/-^*; 6 female/7 controls; 4 female), 4 months old (9 *Cstb^-/-^*; 5 female/6 controls; 2 female) and 6 months old (9 *Cstb^-/-^*; 8 female/4 controls; all female) were used.

A TBSS analysis of the FA in 6-month-old *Cstb^-/-^* and control groups (without hemisphere mirroring) has been previously published together with TBSS data from EPM1 patients [9]. As the observed changes were bilateral, hemisphere-mirrored FA-maps were used in the current analysis as described previously [16]. The previously measured data from 6-month-old animals [9] were re-analysed using hemisphere mirroring and included as the final time point in this study.

The *ex vivo* DTI was performed as previously described [9]. Briefly, 4% paraformaldehyde fixed brains were immersed in perfluoroether (Fomblin, Solvay Solexis, Milan, Italy) and DTI was performed in a 9.4 T vertical magnet (Oxford Instruments, Abingdon, UK) interfaced to a Varian DirectDrive console (Varian Inc., Palo Alto, USA). Data acquisition was done using a 3D fast spin echo sequence (TR  = 1000 ms; TE  = 28 ms; echo train length  = 2, data matrix 128×64×64, zero padded to 256×128×128; FOV 20×10×10 mm^3^). Six 3D data sets with diffusion weighting (diffusion time 17 ms, b-value 1000 s/mm^2^) in six non-collinear directions and one data set without diffusion weighting were acquired. The measurement time was 16 hours.

### 2.5 Tract-Based Spatial Statistics

For TBSS analysis we utilized the TBSS protocol for FA [14] modified for use in rodents [16]. The TBSS analysis of FA-values comparing *Cstb^-/-^* mice with controls was performed for all time points (2, 4 and 6 months). In order to present all TBSS results in a common 3D space, thus making comparison of affected WM-tracts easier, all 44 animals were also registered into a single best registration target. In brief, the diffusion tensor model for each brain was calculated and resulting individual FA maps were used in subsequent co-registration and TBSS procedures. To be able to co-register all animals in the study to a common 3D space, a free-search for the most representative brain, and thus the best registration target in the whole data set, was applied. This was done in order to minimize the image warping required for each brain volume. The calculated best target was subsequently used in TBSS-scripts as a template into which final transformations were targeted to. After target selection, direct non-linear co-registration of individual FA maps was then applied to this common template brain. Hemisphere mirrored FA-maps were included in the analysis and treated as repeated measurements [16]. We also assessed whether changes in axial (AD, diffusion along the axonal tract) or radial (RD, radial diffusion perpendicular to axons) diffusivity were responsible for the FA changes. AD and RD parameters were brought to the same 3D space using the co-registration warp-fields and tract projection information obtained during FA TBSS processing. Finally, the results of the TBSS analysis with p<0.05 (Threshold-Free Cluster Enhancement with multiple comparison correction) were deemed significant. FA values in selected anatomical areas were extracted by manually determining the ROI along the FA-skeleton. Significant FA TBSS findings and mouse brain atlas [15] were used for navigation. The extracted values of the *Cstb^-/-^* group were compared to those of the control group. For statistical comparisons, we performed one-way ANOVA with Bonferroni post hoc correction using GraphPad (Prism 5.0c; GraphPad Software, La Jolla, USA) and considered p<0.05 as significant.

## Results

### 3.1 MRI volumetry in Cstb^-/-^ and control mice

**Figure 1:**
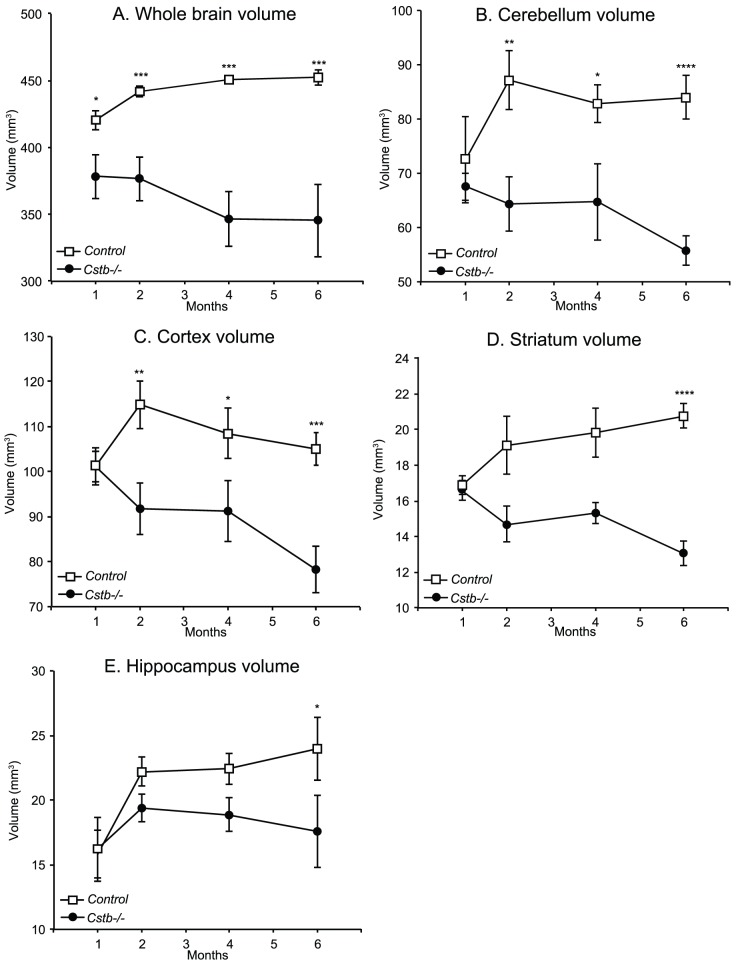
*In vivo* MRI-volumetry of *Cstb^-/-^* mice revealed early and severe brain atrophy. *In vivo* MRI-volumetry of *Cstb^-/-^* mice from 1 to 6 months showed differences in the volumes of A) the whole brain (excluding the medulla), B) cerebellum, C) cerebral cortex, D) striatum and E) hippocampus. Time points showing a significant difference in volume are marked with asterisks (statistical comparison was done using one-way ANOVA with post hoc correction and with p<0.05 considered significant; *, p<0.05; **, p<0.01; ***, p<0.001; ****, p<0.0001).

Using *in vivo* MRI volumetry, we measured volumes of the whole brain, cerebellum, cortex, striatum and hypothalamus at 1, 2, 4, and 6 months of age in individual animals. The whole brain volume, quantified excluding the medulla, was significantly smaller in *Cstb^-/-^* mice compared to controls already at 1 month, and brain atrophy was extensive in the 6-month-old animals (Fig. 1 A). While the whole brain volume in controls continued to increase until 4 months, it decreased in *Cstb^-/-^* mice from 2 months onwards (Fig. 1 A). At one month, there was no difference in cerebellar volume between controls and *Cstb^-/-^* mice (Fig. 1 B). The cerebellar volume in control mice increased until 2 months of age, whereas in *Cstb^-/-^* mice, the cerebellar volume progressively decreased from 1 month until 6 months of age and showed a statistically significant difference from 2 months onwards. Progress of the volume loss in the cerebral cortex (Fig. 1 C) was similar to that in the cerebellum, with *Cstb^-/-^* mice showing a significant decrease from 2 months onwards. The striatum and the hippocampus (Fig. 1 D and E) showed significant reductions in volume later, at 6 months of age. It is worth noting that the difference in volume between the groups was accentuated over the study period as the brain volume in the control group continued to grow, whilst in the *Cstb^-/-^* group, the brain volume decreased from 1 month onwards.

### 3.2 TBSS comparison of DTI data between Cstb^-/-^ and control mice

**Figure 2:**
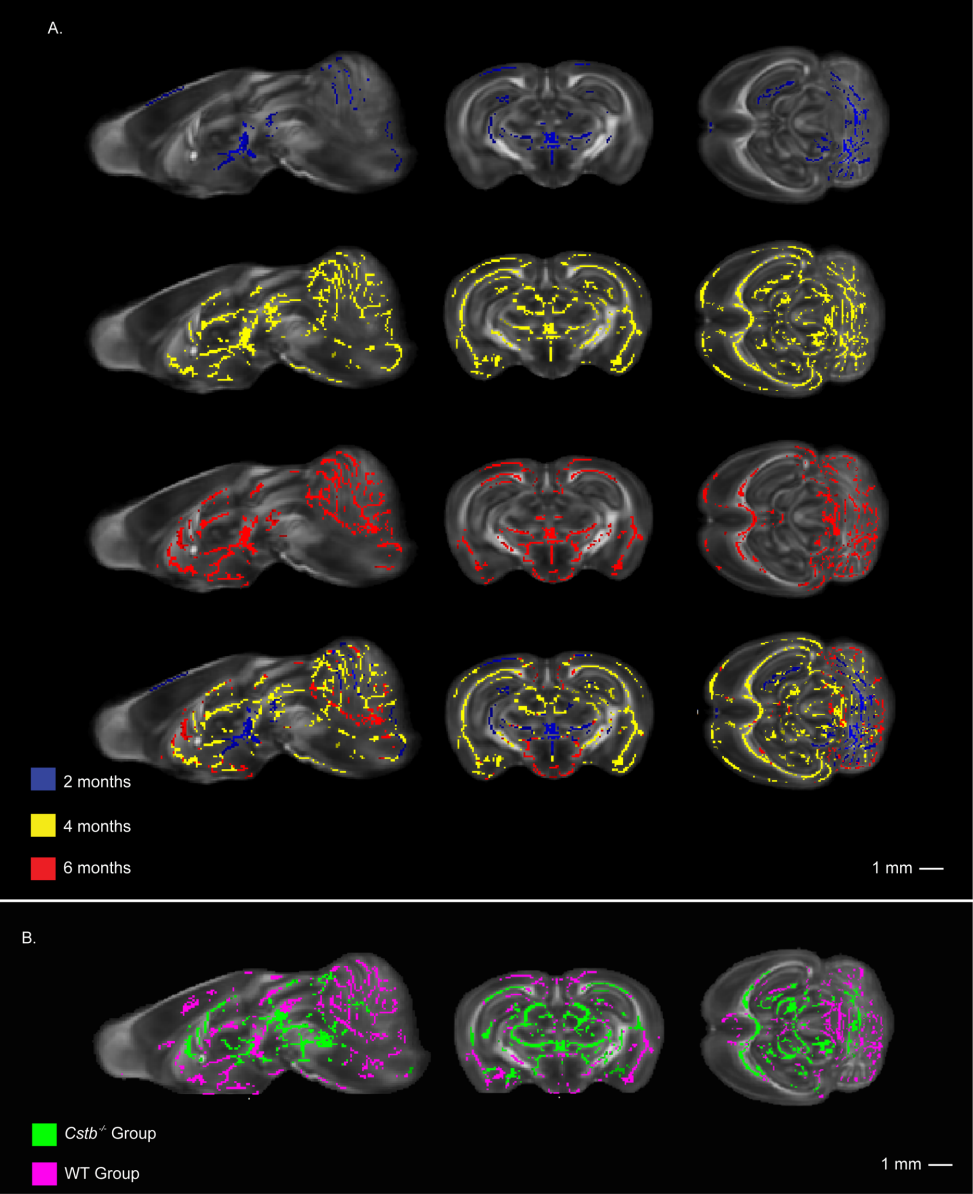
TBSS analysis of DTI data showed WM-changes emerging at the thalamus and cerebellum. A) The TBSS comparison of FA between *Cstb* ^-/-^ mice and controls shown in three orientations: sagittal (left), coronal (middle) and horizontal (right). Areas of significant (p<0.05, multiple comparison corrected, with threshold-free cluster enhancement) FA decrease are shown in blue at 2 months of age (first row), yellow at 4 months of age (second row) and red at 6 months of age (third row), and a composite image overlaying all timepoints (fourth row). B) Longitudinal TBSS analysis of the FA between 2 months and 6 months of age show areas of significantly lower FA at 2 months compared to 6 months of age. The green color represents areas of significant FA change in *Cstb^-/-^* mice and pink shows control mice, with the *Cstb^-/-^* group results on top. Results are shown layered on top of the mean FA map calculated from all brains in the study. The FA changes are shown in three orientations: sagittal (top), coronal (middle) and horizontal (bottom).

TBSS analysis of the DTI data revealed a statistically significant decrease in FA in several brain regions in *Cstb^-/-^* mice compared to the controls at 2 months (Fig. 2 A; blue color). These regions include the cerebellum, cerebral cortex, external/internal capsule, hippocampus, hypothalamus, and thalamus, with the most dramatic changes in the cerebellum and the thalamus. At 4 months (Fig. 2 A; yellow color), more widespread decrease in FA within the cerebellum, cerebral cortex, external/internal capsule, hippocampus, hypothalamus, and the thalamus was detected. At this time point, TBSS also revealed decreased FA in many brain regions not affected at 2 months, including the corpus callosum, medulla, and the striatum. Finally, at 6 months (Fig. 2 A; red color) the decrease in FA was seen in larger areas within the previously affected regions, most notably in the corpus callosum, hypothalamus, thalamus, and cerebellum. However, at 6 months of age, only minor changes were detected in the hippocampus. The TBSS analysis detected significantly elevated RD in the thalamus and the cerebellum of the *Cstb^-/-^* group at 4 months and in the cerebellum at 6 months (data not shown). TBSS analysis of AD did not reveal significant differences in any time-point.

### 3.3 TBSS analysis of DTI data within Cstb^-/-^ mouse and control groups

The data also showed that FA in the WM changed as the animals aged and the WM matured (Fig. 2 B, Table 1). TBSS analysis in the control group showed that the FA-values in most WM tracts at 2 months were significantly lower when compared to values at 6 months (Fig. 2 B; FA change in the control group from 2 to 6 months in pink color, bottom layer), demonstrating FA increase in the WM as a normal phenomena in aging animals. Similar changes in FA values were also observed in the *Cstb^-/-^* group (Fig. 2 B; FA change from 2 to 6 months in the *Cstb^-/-^* group in green color, top layer), although significant FA changes were detected in fewer brain regions. Also, in some brain regions such as the hypothalamus, thalamus and cerebellum, changes were detected only in restricted sub-regions.

**Table 1:** ROI based quantification of FA values in different anatomical locations in *Cstb^-/-^* and control mice.

	FA 2 Months	FA 4 Months	FA 6 Months
	*Cstb* ^-/-^/control	*Cstb* ^-/-^/control	*Cstb* ^-/-^/control
**Amygdala**	0.25±0.01/0.27±0.01	0.26±0.01/0.28±0.01	**0.29±0.01/0.32±0.01**
**Brainstem**	0.41±0.01/0.43±0.01	0.42±0.01/0.45±0.01	**0.46±0.01/0.51±0.02**
**Cerebellar peduncle**	0.48±0.01/0.48±0.01	0.53±0.02/0.52±0.01	0.53±0.02/0.54±0.01
**Cerebellum**	**0.47±0.01/0.55±0.03**	**0.46±0.01/0.62±0.01**	**0.46±0.03/0.59±0.04**
**Corpus callosum**	0.54±0.03/0.56±0.06	0.52±0.02/0.63±0.05	**0.56±0.03/0.74±0.04**
**Cortex**	**0.24±0.01/0.30±0.02**	**0.22±0.01/0.31±0.02**	**0.24±0.01/0.29±0.01**
**External capsule**	0.37±0.01/0.36±0.01	**0.36±0.01/0.41±0.01**	**0.34±0.01/0.43±0.01**
**Hippocampus**	0.39±0.01/0.42±0.02	0.39±0.01/0.43±0.01	**0.45±0.02/0.53±0.01**
**Hypothalamus**	0.30±0.01/0.33±0.02	0.34±0.01/0.35±0.02	**0.31±0.01/0.40±0.02**
**Internal capsule**	0.63±0.01/0.66±0.01	**0.61±0.01/0.68±0.01**	**0.58±0.01/0.64±0.02**
**Striatum**	0.27±0.01/0.27±0.01	0.24±0.01/0.28±0.02	0.20±0.01/0.23±0.03
**Thalamus**	**0.36±0.02/0.43±0.02**	**0.37±0.01/0.44±0.01**	**0.44±0.01/0.54±0.03**

Values have been averaged from hemisphere-mirrored FA-skeletons. Brain regions showing significant (statistical comparison was done using one-way ANOVA with Bonferroni post hoc correction and with p<0.05 considered significant) differences in FA values are in bold. All values are shown with the standard error of mean (SEM).

## Discussion

Previous imaging studies of EPM1 patients have demonstrated atrophic changes in GM and WM in several brain regions, most notably in the cortices and the thalamus [8], [9], [17]–[20]. The diversity in anti-epileptic drug treatments, as well as the heterogeneity of the studied patient groups in terms of age, gender and disease duration have made interpretation of the patient data difficult and thus the existing reports offer only a limited view of EPM1 pathology. Longitudinal imaging studies in human patients are not usually feasible as they are expensive to perform and in a disease like EPM1, could span decades. Thus, the progression of pathological changes in EPM1 has remained unclear. Since previous studies have implicated that the *Cstb* ^-/-^ mouse is a good model for EPM1 in terms of clinical features [10], and brain pathology [9], [13], it provides a mean to monitor the spatiotemporal progression of brain pathology with implications to disease course in human patients as well.

**Figure 3:**
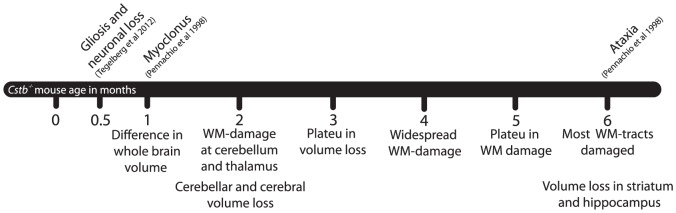
Sequence of pathological events in *Cstb* ^-/-^ mice. The figure summarises the sequence of the pathological events in the *Cstb* **^-/-^** mice as detected in the current study. Emergence of key symptoms and previous findings reported by Pennacchio et al. (1998) and Tegelberg et al. (2012) have also been included in the figure.

Here we performed a study in *Cstb^-/-^* mice utilizing DTI accompanied by TBSS analysis and longitudinal MRI volumetry to investigate the spatiotemporal pattern of atrophy and WM damage from early symptomatic (1 month of age) to fully symptomatic (6 months of age) mice. The time points were selected based on our previous histological data [13] and manifestation of disease symptoms in mice [10]: at 1 month of age the mice show glial activation, neuron loss in the cerebral cortex and the first signs of myoclonia, and at 6 months of age the mice usually demonstrate ataxia accompanied by widespread gliosis and neuron loss in the brain (Fig. 3). Our results show that in the *Cstb^-/-^* mouse brain also the WM is affected early in disease pathogenesis and is progressively damaged, eventually affecting all major WM tracts. The brain atrophy and WM damage manifest with varying degree and timing in different brain regions, the cerebellum and the thalamus being among the most vulnerable regions. Importantly, our study demonstrates that a multi-timepoint study of DTI changes combined with TBSS analysis is a feasible approach both for investigating progressive WM damage in neurodegenerative mouse models and for detecting FA changes related to normal WM maturation and reorganisation.

Cerebellar [18], thalamic and cortical atrophy [8] has been reported in EPM1, and the whole brain volume in adult EPM1 patients has been shown to be 10% smaller compared to healthy controls [8]. In agreement, limited histopathological data on EPM1 patients have shown loss of cerebellar granule and Purkinje cells as well as neuronal degeneration and loss in the cerebral cortex, striatum, thalamus and brain stem [1], [21]–[23]. Although the progressive nature of atrophy in EPM1 patients has been difficult to demonstrate, the severity of myoclonia has been shown to be associated with cortical thinning [8]. The histopathological findings in the *Cstb^-/-^* mice are very similar to those in EPM1 patients, although atrophy is more drastic in the mouse brain [13]. Studies from young adult *Cstb^-/-^* mice have shown volume loss and selective loss of neurons within the cerebellum and cerebral cortex and the relative sparing of the hippocampus [10], [12], [13], [24]. Alterations in GABAergic signalling have also been demonstrated both in young and aged animals [25], [26]. Our volumetric data are concordant with these findings as the MRI data in the *Cstb^-/-^* mouse brain show that the volume loss, which is widespread already at the early stages of disease, is not spatially uniform and is progressive with only limited WM damage and late atrophy in the hippocampus. Moreover, the data show that in *Cstb^-/-^* mice the brain volume never reaches that in the control mice. Even though we did not detect statistically significant atrophy of individual brain regions in one month-old animals, the total brain volume was significantly smaller already at this point. Therefore, it is possible that disturbed developmental processes precede neurodegeneration in *Cstb^-/-^* mouse brain [10], [12], [13].

We have previously shown that WM damage in 6-month old *Cstb^-/-^* mice reflects local axonal degeneration [9]. The sequence and timing of pathological changes in the *Cstb^-/-^* mouse brain imply that the WM damage is most likely secondary to the early-onset non-uniform microglial activation that precedes astrocytic activation, neuron loss and the appearance of myoclonus [13]. Activation of microglia and secretion of proinflammatory cytokines are known to contribute to many neurodegenerative disorders, including Alzheimer's disease, Parkinson's disease, amyotrophic lateral sclerosis and Huntington's disease [27]. Moreover, microglia-derived cathepsin B, a target protein of CSTB, has been shown to have a role in WM damage in a mouse model of multiple sclerosis [28]. Cathepsin B levels are increased in both *Cstb^-/-^* mice and EPM1 patient cells [29], [30] and mice deficient for both CSTB and cathepsin B show significantly less cerebellar apoptosis [31]. Thus, it is possible that elevated cathepsin B activity due to reduced inhibition by CSTB contributes to axonal degeneration and subsequent WM loss also in *Cstb^-/-^* mice.

The *Cstb^-/-^* mice showed a decrease in FA on several brain regions already at 2 months of age with the changes accentuating as the animals aged. This finding suggests that in the future, longitudinal DTI combined with TBSS could be a feasible approach for monitoring progressive damage of the WM in mouse models. Decreased FA in *Cstb^-/-^* mice presumably is a consequence of axonal degeneration, since loss of axons results in higher extracellular volume in the WM, which increases radial diffusion of water parallel to tracts. This is reflected as increased RD in DTI, which became evident in the 4-months -old *Cstb^-/-^* mice in our study. As TBSS analysis of axial diffusion did not reveal significant differences at any time-point, it is probable that an increase in radial diffusion contributes more to the detected decrease in FA than the changes in axial diffusion.

The WM matures as the animals age and in healthy mice, increasing fibre tract coherence and compaction in the WM can be detected as a FA increase. The increase in FA has been shown to continue well beyond 2 months of age [32]–[34]. In agreement with these previous results, our TBSS analysis of DTI data in the control mouse group showed increased FA values in most major WM tracts from 2 to 6 months of age. The data thus demonstrate that TBSS could also be utilized to monitor maturation of healthy WM. In the *Cstb^-/-^* mice, a less pronounced FA increase over time was observed in the most seriously damaged regions, e.g. cerebral cortex, cerebellum and thalamus, probably illustrating the concurrent degenerative changes.

EPM1 is characterized by stimulus-sensitive myoclonus, tonic-clonic seizures and ataxia. While epileptic seizures in EPM1 can be well controlled with drugs, myoclonus is in most cases highly incapacitating and remains resistant to therapy. The myoclonus is often the manifesting symptom in EPM1 patients and becomes progressively more debilitating over the next 5 to 10 years followed by stabilization [35], the reason for which is unknown. EPM1 patients share clinical characteristics with subcortical movement disorders that manifest as ataxia and myoclonus from subcortical structures. Previous studies have shown altered inhibition in the motor cortices and the thalamocortical system of the patients [36]–[38] and a thalamocortical dopaminergic defect in EPM1 has also been reported [39]. In addition, we recently reported widespread WM damage in EPM1 patients [9]. Concordantly, previous [9], [13] and current results from mice show subcortical structures involved in movement control (e.g. striatum and thalamus) severely affected. The present data from *Cstb^-/-^* mouse also confirms that the pathological changes in the WM are widespread and that the changes manifest early, are progressive and show regional specificity. Based on the extent and temporal pattern of pathologic changes in the cerebral cortex, thalamus and cerebellum in EPM1 patients and *Cstb^-/-^* mice [9], [13] it is likely that atrophy and WM damage in the thalamocortical system and the cerebellum both contribute to the motor disturbances in EPM1 and explain their resistance to treatment.

## Conclusions

The brain pathology of *Cstb^-/-^* mice is characterized by early-onset and progressive degeneration affecting all major WM tracts, which likely occurs secondary to glial activation and neuronal death and probably augments the myoclonic seizures and ataxia. A multi-timepoint study of DTI changes with TBSS analysis provides a feasible approach to follow both normal WM development and reorganisation and progressive WM damage in mouse brain.
